# Determinants of malaria transmission in Indian districts in 2018: insights from ensemble models

**DOI:** 10.1186/s12936-025-05546-9

**Published:** 2025-10-14

**Authors:** Avik Kumar Sam, Alisha Khatoon, Harish C. Phuleria

**Affiliations:** 1 Environmental Science and Engineering Department, Indian Institute of Technology Bombay, Mumbai, India; 2 Center for Climate Studies, Indian Institute of Technology Bombay, Mumbai, India; 3 National Disease Modelling Consortium, Indian Institute of Technology Bombay, Mumbai, India

**Keywords:** Malaria, Land use, Land cover, Weather, Socio-economic inequities, Policy, Machine learning

## Abstract

**Background:**

The National Framework for Malaria Elimination, formulated in 2016, aims to eliminate malaria in India by 2030, focusing on the districts as the strategic units for planning and implementing intervention measures. In this study, the spatial distribution of malaria hotspots was investigated and the significant covariates were identified that are potentially influencing its transmission in India.

**Methods:**

District-wise data on malaria, socio-economic variations, meteorological factors and land-use land-cover changes were used to assess the impact of covariates on the transmission using an ensemble of Random Forest and Poisson regression models.

**Results:**

Spatial clusters for annual parasite incidence (API) and annual falciparum incidence (AFI) were observed distinctly in the central and northeastern districts, but additional hotspots for non-AFI transmission were present in the northern districts. The ensemble models suggest that in districts with high malaria transmission, socio-economically marginalized communities with water inaccessibility and not having a mobile phone are among the potentially vulnerable sections of the society. The use of unprotected water source for household consumption is also potentially linked to increased transmission. Mother’s education could reduce malaria transmission, but a substantial proportion of the mothers in high-transmission districts reporting API > 1 did not have any formal or informal education.

**Conclusions:**

Socio-economic development that includes concerted efforts to impart both technical and non-technical education and spreading awareness for better lifestyle choices will help reduce the malaria burden in the country. The clusters of high-burden districts instead of individual districts are recommended as strategic points for implementing targeted interventions.

**Supplementary Information:**

The online version contains supplementary material available at 10.1186/s12936-025-05546-9.

## Background

Malaria continues to be a major public health challenge with high morbidity and mortality rates [1]. Eradicating malaria permanently has been the overarching goal of the global community [2], with the World Health Assembly endorsing a 90% reduction in malaria burden by 2030 [3]. In India, the National Framework for Malaria Elimination, aligning with the World Health Organization’s Global Technical Strategy and the Malaria Elimination Roadmap of the Asia Pacific Leaders’ Malaria Alliance 2016–2030, was formulated by the National Centre for Vector Borne Disease Control Programme (NCVBDC) Programme in 2016 [4]. Regional malaria control and the development of necessary health systems are given importance, while districts are focused as the strategic point for planning and implementing intervention measures [4].

Multiple studies have indicated that malaria incidence is significantly influenced by the prevailing climatic variations and socio-economic inequities [1, 5, 6]. Further, changes in land use and land cover are also associated with malaria risk [7, 8]. Understanding their role in malaria transmission can greatly help eradicate malaria [1, 9]. Previously, development programmes were advocated as essential for malaria control [1]. In Europe, malaria declined with socio-economic development, involving the modernization of livestock production and farming [10].

In India, malaria cases and deaths have declined significantly in the last decade: 1018 deaths in 2010 decreased to 96 in 2018. However, there are multiple challenges, such as a few endemic regions where the decline in malaria incidence is much lower than the national average [4]. Multiple region-specific studies in India have proved a relationship between malaria, socio-economic factors, and climatic parameters [11–13]. The stakeholders have also identified the necessity of the eliminations targeted in a phased manner, accounting for the differences in their socio-economic conditions and eco-epidemiological settings [4]

Previously, in Africa, millions of blood-fed mosquitoes reportedly migrated over hundreds of kilometres, thus spreading malaria across their routes [14]. Thus, the interventions should not be implemented in a particular endemic district only, as there is a risk of malaria transmission due to the migratory mosquitoes and the population [15]. Therefore, conducting a systematic analysis at the national level is essential. The present study aims to assess the spatial distribution of malaria transmission across the districts. The endemic spatial clusters are also identified for all the malaria parameters. The impact of socio-economic inequities, land-use land cover changes and meteorological variations is investigated through an ensemble of classical Poisson and Random Forests regression models.

## Methods

### Disease data

Malaria prevalence data from 2018 for 635 districts in India were obtained from the NCVBDC, which is the central nodal agency under the Ministry of Health and Family Welfare, Government of India that administers a programme for the prevention and control of malaria. The epidemiological variables for which the data were obtained and their formulae are provided below:

1. Percentage of falciparum cases (% PF):
  $$PF = \frac{Number\;of\;cases\;from\;P.\;falciparum}{{Total\;malaria\;cases\;reported\;in\;2018}} \times 100$$
2. Annual blood examination rate (ABER):
  $$ABER = \frac{Number\;of\;blood\;smears\;collected\;in\;2018}{{Population\;under\;surveillance}} \times 100$$
3. Annual parasite incidence (API):
  $$API = \frac{Malaria\;incidence\;in\;2018}{{Population\;under\;surveillance}} \times 1000$$
4. Slide positivity rate (SPR):
  $$SPR = \frac{Number\;of\;blood\;smears\;positive\;for\;a\;malaria\;parasite}{{Total\;blood\;smears\;examined}} \times 100$$
5. Annual falciparum incidence (AFI):
  $$AFI = \frac{Number\;of\;cases\;from\;P.\;falciparum}{{Total\;malaria\;cases}} \times 100$$
6. Slide falciparum rate (SFR):
  $$SFR = \frac{Number\;of\;blood\;smears\;positive\;for\;P.\;falciparum}{{Total\;blood\;smears \;examined}} \times 100$$
7. Deaths reported due to *Plasmodium vivax.*
8. Deaths reported due to *Plasmodium falciparum.*

### Covariates

Covariates used in the study include socio-economic, land-use land-cover, and environmental and meteorological variables. Socio-economic data were obtained from the National Family Health Survey conducted in 2019–2021 [16]. Household data on indicators covering health, wealth, nutrition, population and household characteristics, and healthcare accessibility were aggregated to the unit districts for the analysis using SPSS version 28.0.0.0 (190) and Python version 3.10. Satellite data on land-use land cover were obtained from the ESA Sentinel-2 imagery [17]. The administrative boundaries of the districts were superimposed on the gridded data of land use land cover (LU/LC) classes such as water bodies, forest, croplands, flooded vegetation, bare and built-up areas and snow cover, spread over the geographical extent of India at a 10 m resolution using ArcMap version 10.6. Environmental and meteorological variables like temperature, precipitation, specific humidity (SH), and Particulate Matter were obtained from Modern-Era Retrospective Analysis for Research and Applications, version 2 (MERRA-2) satellite reanalysis data from NASA [18], and agglomerated to the districts using Geopandas version 0.14.3. These datasets were subjected to robust quality control and quality checks, after which summarisation was performed.

### Clustering using Getis-Ord

The clusters in the distribution of malaria-specific epidemiological data variables were assessed using the Getis-Ord Gi* statistic that compares the global mean malaria cases of all districts with the local mean malaria cases of each district and neighbouring districts [19]. Getis-Ord Gi* statistic is defined as

$$G_{i} \left( d \right) = \frac{{\mathop \sum \nolimits_{j = 1}^{n} w_{ij} \left( d \right)x_{j} }}{{\mathop \sum \nolimits_{j = 1}^{n} x_{j} }},\quad j \ne i$$

where ${x}_{j}$ denotes the attribute value of an event $j$, ${w}_{ij}$ represents the spatial weight of $i$ and $j$, and n is the total number of features. Districts with standardized z-score > 0 were classified as hotspots. P-values were used to reject null hypothesis and accept alternate hypothesis, which states that spatial clustering exists. Arc Map version 10.6 and ESDA version 2.4.3 were used for all spatial analysis.

### Bivariate association

Spearman’s Rank correlation test was performed to identify the critical covariates that could affect malaria transmission in the districts. API was used as the outcome variable. Spearman’s Rank correlation is a non-parametric statistic that measures the statistical dependence between ranked variables using a monotonic function [20]. The Spearman correlation coefficient is computed using the formula:

$$r_{s} = 1 - \frac{{6 \sum d_{i}^{2} }}{{n\left( {n^{2} - 1} \right)}} , d_{i} = R\left( {X_{i} } \right) - R\left( {API_{i} } \right)$$

where $i$ is the observation of ‘$n$’ total observations and $X$ denotes the variable. The bivariate associations were computed using Scipy version 1.13.0 [21].

### Ensemble modelling

The significant variables (p-value < 0.05) identified from the Spearman correlation were subjected to fit ensembles of Random Forest (RF) models. Based on model aggregation, RF combines multiple binary decision trees developed through bootstrapping [22]. These models have been extensively used for ranking the importance of a variable, also referred to as ‘variable importance’, in explaining the variability in the outcome variables [22, 23]. For the present study, 75% of the districts was used for training the model and 25% of the districts as test data for estimating the root mean squared error (RMSE). Prior to training, the entire dataset was normalized using a Robust Scaler approach [24]. It is described as

$$x = \frac{{x_{i} - Q_{1} \left( x \right)}}{{Q_{3} \left( x \right) - Q_{1} \left( x \right)}}$$

where ‘x’ is the value for a variable, Q_1_ and Q_3_ represent the 25th and 75th quantile of the variable. The RF models were trained using different combinations of hyperparameters and the best model was selected using the minimum RMSE. The RMSE is given by:

$$RMSE = \sqrt {MSE} \;{\text{where}}\;MSE = \frac{1}{N} \mathop \sum \limits_{i = 1}^{N} \left( {y_{i} - \check{y}_{i} } \right)^{2}$$

where *y*_*i*_ and $\check{y}_{i}$ are the actual and predicted API, respectively. To ensure the stability and robustness of the selected model, a Monte Carlo simulation was performed involving 100 random independent iterations that generated a matrix of ranked variables and their variable importance. These were then aggregated to identify the most significant variables that explain the variability in the API across the districts. The variability in these variables across the districts is provided in Supplementary Table S1. As 16% of the districts reported zero API, a zero-inflated Poisson (ZIP) Regression approach was used [25]. Prior to ZIP modelling, the API per 10,000 people was computed and the union territories of Daman and Diu and the Islands, Lakshadweep and Andaman and Nicobar Islands were removed after data quality checks.

**Fig. 1:**
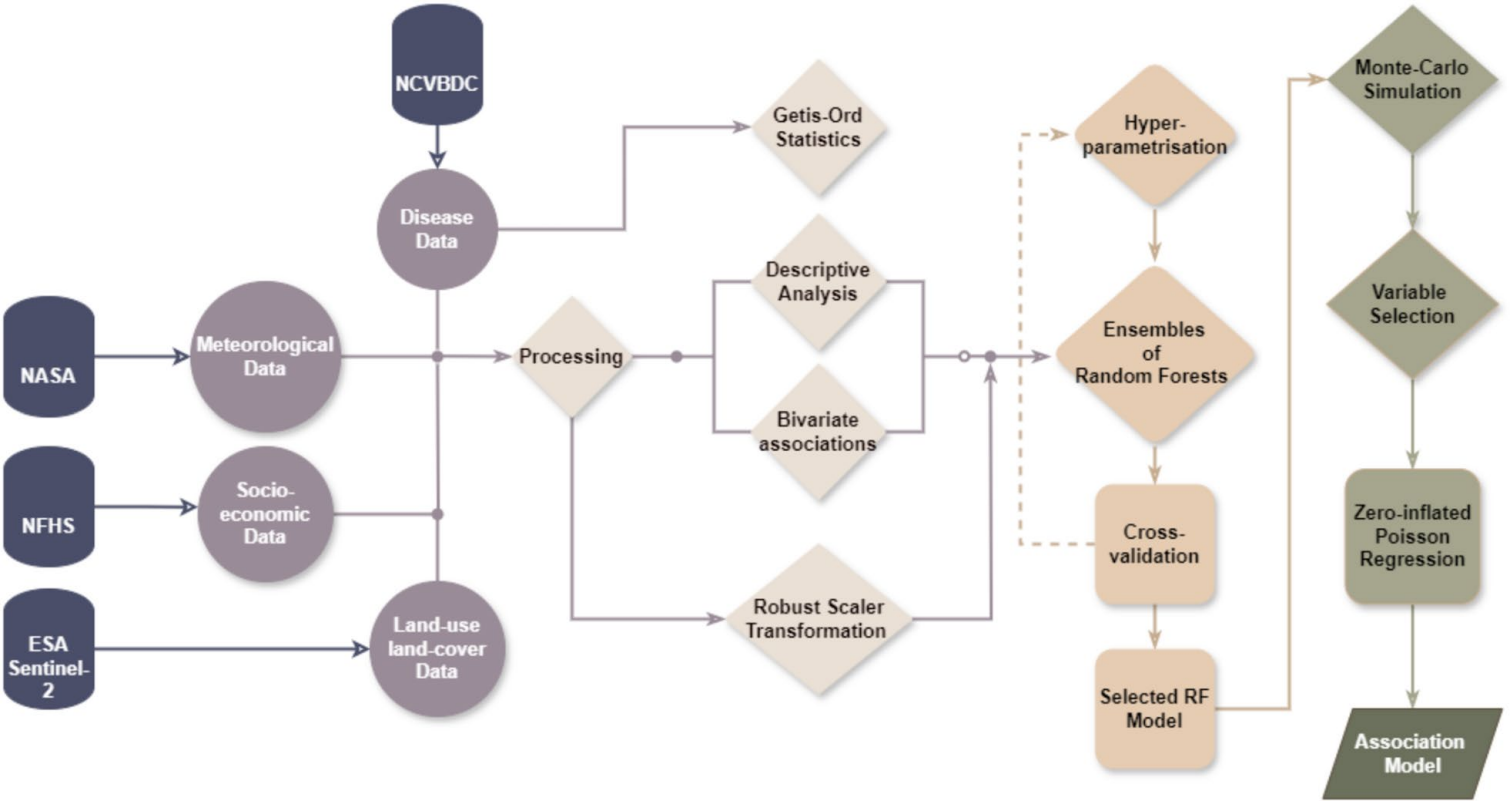
Methodology framework illustrating the inputs, data processing methods and outputs

Unlike traditional generalized linear modelling, the ZIP has two components: it models the counts using a Poisson distribution, while a logistic regression model is used to estimate the probability of an observation being zero. The two components are then combined for the cumulative probability mass function (*pmf*). Mathematically, the *pmf* is given by:

$$P\left( {API = y} \right) = \left\{ {\begin{array}{*{20}c} {p + \left( {1 - p} \right)e^{{ - {\uplambda }}} ,} & {y = 0} \\ {\left( {1 - p} \right)\frac{{e^{{ - {\uplambda }}} {\uplambda }^{y} }}{y!},} & {y \ge 0} \\ \end{array} } \right.$$

where $p$ represents the probability of a district having 0 API and $\uplambda$ denotes the rate parameter of the Poisson distribution that depends on covariates through a logit link function. The ZIP model allows identification of the significant variables that could potentially influence malaria transmission for both types of districts: districts with zero API and those with API $\ge$ 0. The exponentiated risk of association, i.e., the risk ratio (RR) for the Poisson model and the odds ratio (OR) for the logistic model, were also estimated. A categorical variable ‘transmission’ based on the *Plasmodium falciparum* transmission was introduced to differentiate between *Plasmodium vivax* and *P. falciparum*. The Python libraries, Statsmodels (v. 0.13.2) and Sklearn (v.1.1.2), were used for the ensemble modelling discussed above. The entire methodology is summarized in Fig. 1.

## Results and discussion

### Distribution of malaria across India

**Fig. 2:**
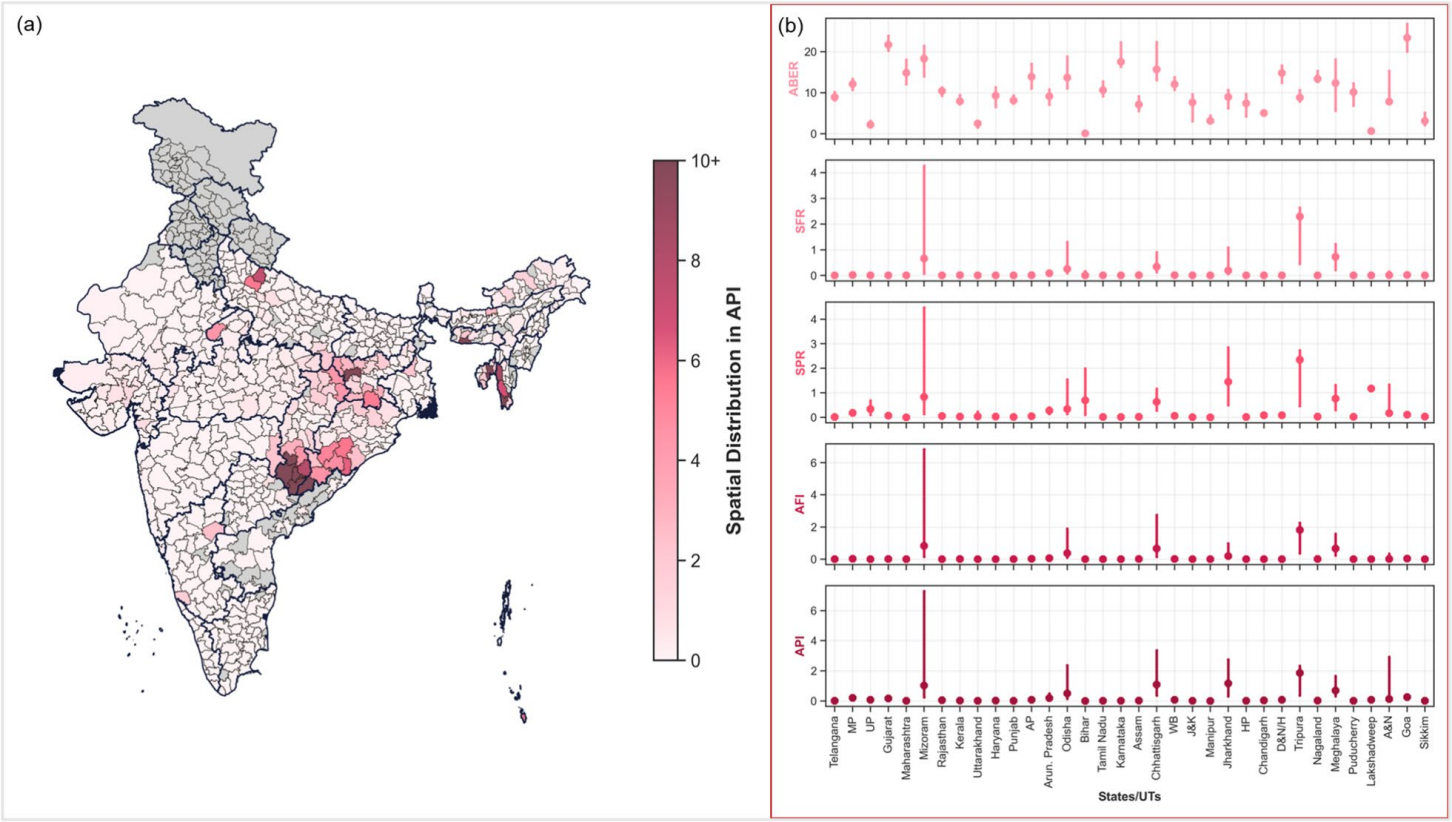
**a** Spatial distribution of API across the districts. **b** Variability in the malaria parameters across the states. The error bar represents the interquartile range; the scatter denotes the median values. The grey colour in **a** indicates data unavailable at the district level. *WB* West Bengal; *D&N/H* Dadra, Nagar Haveli, Daman & Diu; *MP* Madhya Pradesh; *UP* Uttar Pradesh; *J&K* Jammu & Kashmir; *A&N* Andaman & Nicobar Islands; *AP* Andhra Pradesh; *HP* Himachal Pradesh; *Arun. Pradesh* Arunachal Pradesh

India reported 428,848 malaria cases in 2018, with the densely populated northern state of Uttar Pradesh accounting for 20% (86,486). Out of a total of 96 deaths reported, 39 were in Chhattisgarh, where 97.4% of deaths were attributed to *P. falciparum*; the tribal district of Dantewada reported the highest (9). Eight deaths were due to *P. vivax,* out of which Thane and Palghar in Maharashtra reported three and two, respectively, while Kolkata in West Bengal reported two deaths. Further, the northeastern states of Tripura and Mizoram also witnessed significant malaria outbreaks. For instance, Mizoram reported the highest API (3.6) and the AFI (3.3), while the maximum SPR (2.7) and SFR (2.6) were observed in Tripura. Uttar Pradesh had the lowest testing per 100 population (2.3), whereas Gujarat reported the highest (23.3). States reporting > 10,000 cases included Uttar Pradesh, Chhattisgarh, Odisha, Jharkhand, West Bengal, Madhya Pradesh, Gujarat, Tripura, and Maharashtra. The variations in malaria parameters across the states and the spatial distribution of API across the districts are provided in Fig. 2.

Among the districts, a bell-shaped distribution (median < 1) was observed for API and AFI, as explained by the fact that only 10.4% and 6.3% districts reported API and AFI > 1, respectively. In Chhattisgarh, Bijapur (53.1), Sukma (46.5), Dantewada (41.5), and Narayanpur (25.1) reported very high malaria transmission as their API indicates. In the northeast, South Garo Hills (Meghalaya, 29), Dhalai (Tripura, 20.2), and Lawngtlai (Mizoram, 16.2) witnessed major outbreaks. The transmission in these districts was driven by *P. falciparum* as indicated by the high AFI reported, while the % *P. falciparum* also varied between 84–98%. In contrast, *P. vivax* was mostly responsible for transmission in Latehar (Jharkhand; API: 10.9, % *P. falciparum*: 14.1), Bareilly (API: 7.3; % *P. falciparum*: 46.7) and Badaun in Uttar Pradesh (API: 5.5; % *P. falciparum*: 19.8) and in Andaman and Nicobar Islands (API: 5.7; % *P. falciparum*: 11.9).

Additionally, Unakuti and Khowai in Tripura, Kra Ddadi (Arunachal Pradesh), Yanam (Puducherry), Banka (Bihar), Kapurthala (Punjab), and Peddapally, Siricilla and Yadadri in Telangana also reported % *P. falciparum* > 99%. It was observed that districts reporting low transmission (API < 1) had very low % *P. falciparum*. For instance, API < 0.5 was only reported in districts (n = 255) with no *P. falciparum* transmission. Testing was relatively higher in districts reporting increased transmission and surrounding districts, as indicated by the ABER. The spatial distribution of AFI, % *P. falciparum*, ABER, SPR, and SFR across the districts is provided in Supplementary Figures S1, S2 and S3.

### Identification of spatial clusters

**Fig. 3:**
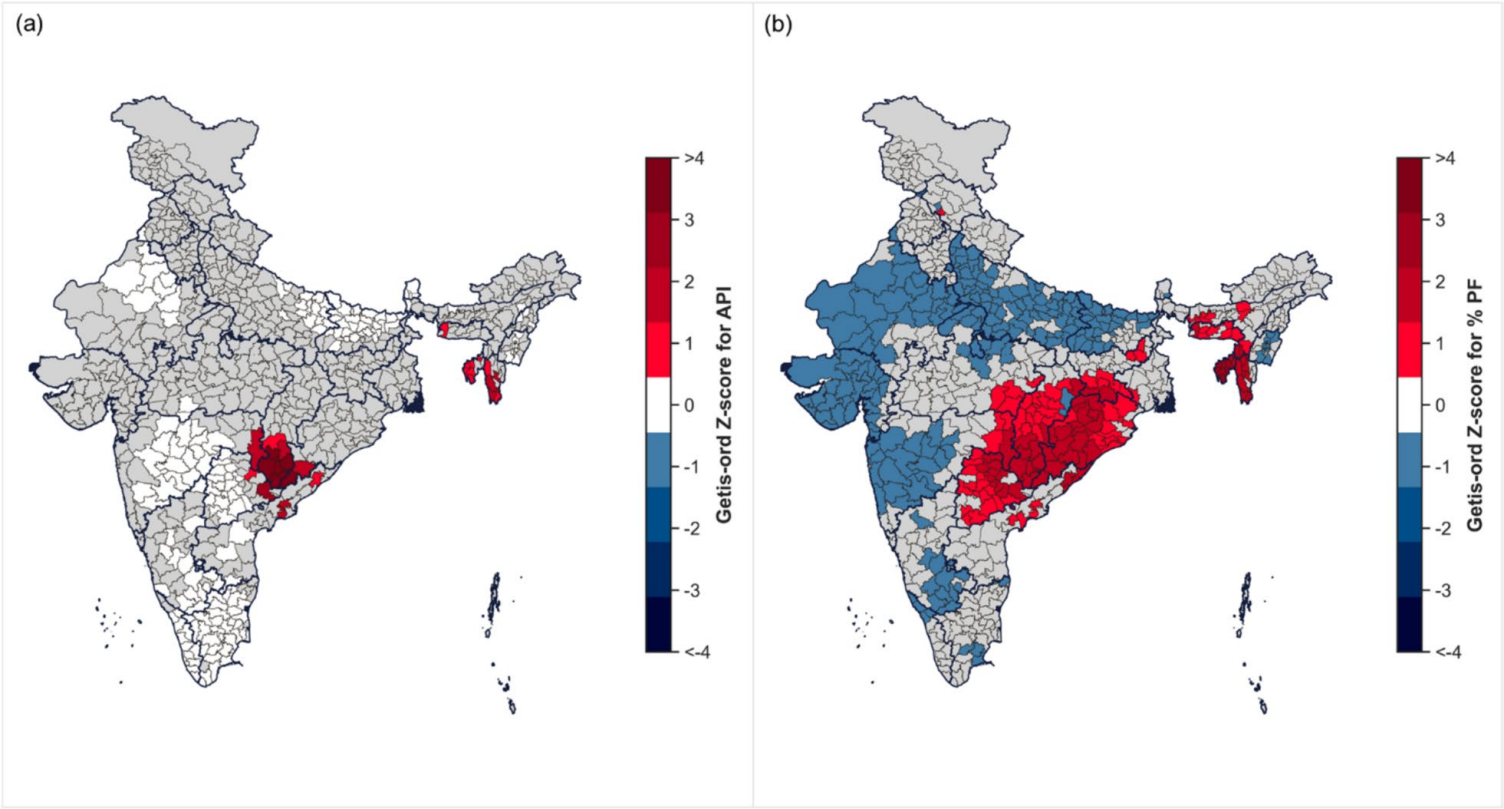
Spatial distribution in hotspots for **a** API and **b** % PF. The grey colour indicates either a non-significant Z-score or data unavailable for the district

Using the Getis-Ord Z-scores and the p-values, districts were classified as hotspots if Z-score > 0 and the p-value < 0.05 [19, 26]. For API, hotspots were identified in Odisha, Andhra Pradesh, Telangana, Chhattisgarh, Jharkhand, and in the northeastern states of Tripura, Meghalaya and Mizoram. The districts for which the Z-score > 2 included Bastar (5.3) and the neighbouring districts of Dakshin Bastar Dantewada (5.2), Sukma (5.2), Bijapur (4.9), Malkangiri (4.5), Narayanpur (3.6), Bhadradri Kothagudem (2.6), Kondagaon (2.2), Gadchiroli (2.2), and Koraput (2.0). In the northeast, Lawngtlai (1.4), Lunglei (1.4), South Tripura (1.0), and Serchhip (1.4) emerged as hotspots. Identical hotspots were observed for AFI with similar Z-scores, except for East Garo Hills and West Khasi Hills in Meghalaya, which were not classified as hotspots for API. The spatial distribution of hotspots for API and % *P. falciparum* is provided in Fig. 3. The hotspots for AFI, SPR, SFR, and ABER are provided in Supplementary Figures S4, S5 and S6.

Similarly, the significant hotspots for % *P. falciparum* was observed in Odisha, Jharkhand, Chhattisgarh, Telangana, Andhra Pradesh, Tripura, Meghalaya, Assam, and Mizoram. Districts with Z-scores ≥ 2 for % *P. falciparum* was mostly concentrated in Tripura, such as Gomati, Khowai, Sipahijala, West Tripura, Dhalai, North and South Tripura, as well as from the neighbouring districts in Meghalaya: Lunglei, Serchhip and Lawngtlai. The hotspots for non-AFI transmission were distributed similarly to API in south-central India. However, few hotspots were found in Uttar Pradesh, Jharkhand, Bihar, and North Chhattisgarh, suggesting the greater involvement of *P. vivax* in these regions. No such clusters were identified in the northeastern states (Figure S7). While Bareilly and Budaun, which had high *P. vivax* transmission, were not recognized as a hotspot for SFR, the neighbouring districts: Rampur, Moradabad, Pilibhit, and Shahjahanpur, were identified as hotspots with moderate clustering (Z-scores: 0.6–0.9). In contrast, hotspots for SPR were concentrated in Bihar: Sitamarhi (1.5), Darbhanga (2.1), Samastipur (2.1), and Begusarai (1.4), but these districts reported very low transmission.

### Bivariate association of covariates with the district-wise malaria transmission

**Fig. 4:**
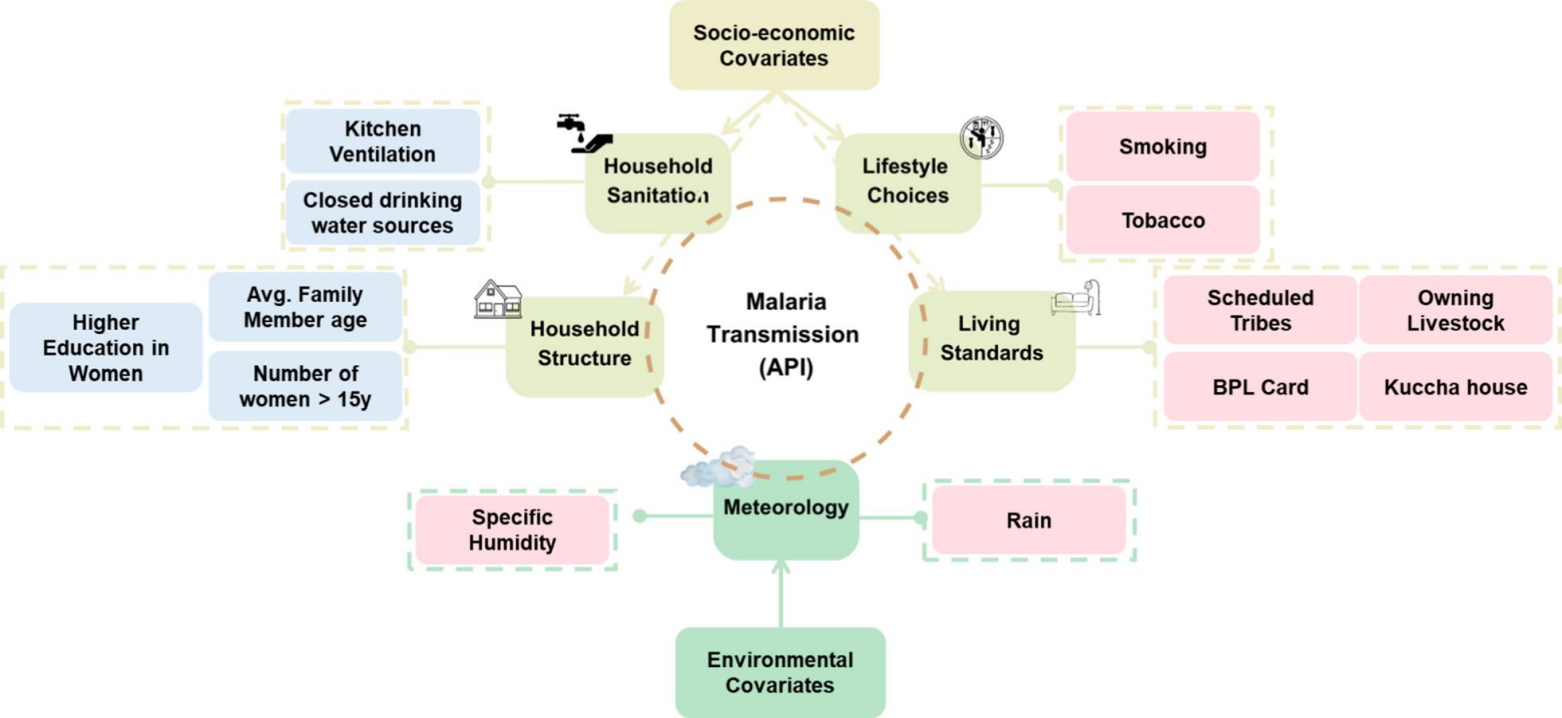
Covariates likely affecting malaria transmission in the districts. Red boxes indicate a positive Spearman association; blue boxes show a negative Spearman association

Spearman’s Rank correlation was used to identify the significant covariates (R2 > 15%; p-value < 0.05) that might influence malaria transmission (API). The significant association shown by API with major socio-economic and environmental covariates is provided in Fig. 4. The district-wise proportion of socio-economic variables describing the household sanitation and structure, as well as lifestyle choices and living standards, was included. Meteorological parameters such as rain and specific humidity as environmental variables were also considered. There was a positive association with tribal communities (Scheduled Tribes) and economically disadvantaged groups, indicated by the population owning a house made of mud/bamboo (Kuccha house) and those belonging to the national benchmark ‘below poverty line’ (BPL Card), as well as the population owning livestock. Education in women and average family age and accessibility to closed sources of drinking water, such as piped water, were negatively associated with API. Previously, backward castes, low education, people residing in kutcha houses or having livestock, and outside water sources, have been linked to an increase in the likelihood of malaria [6, 11, 27].

A negative association between average family member age and API was observed, suggesting that the prevalence of malaria is higher in the younger population. Previously, 30% of cases caused by *P. vivax* were reported in children < 14 years [28] and elsewhere in Sub-Saharan Africa severe malaria attributable to *P. falciparum* was concentrated in younger age groups [29]. Rain and specific humidity were positively associated with API, which was also previously observed in Dehradun, India [30, 31].

### Inferences from the ensemble model

**Fig. 5:**
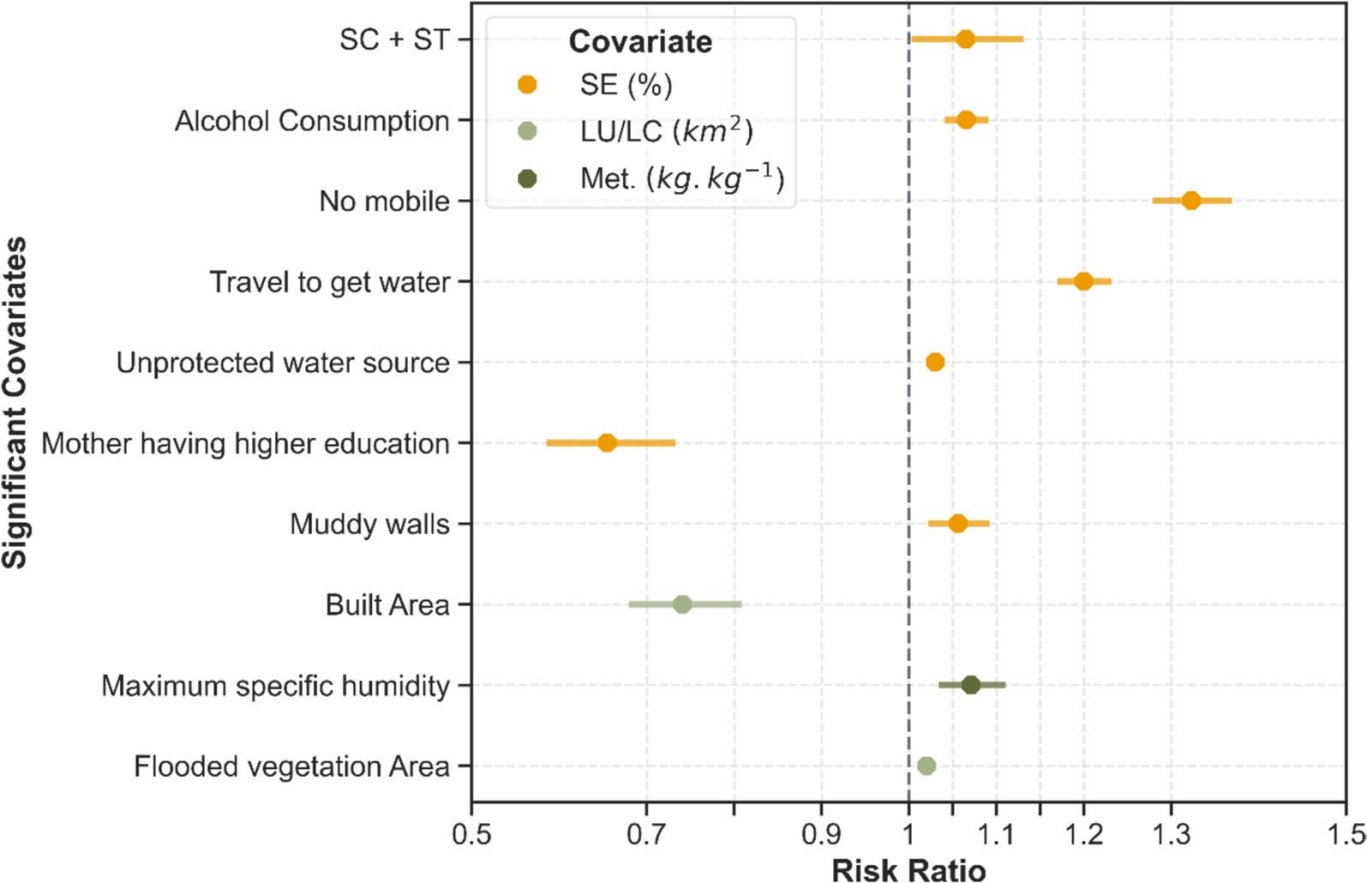
Risk Ratios for the significant covariates estimated from the ensemble RF-ZIP model. The lines represent the 95% Confidence Intervals. *SE* Socioeconomic, *LU/LC* Land-use Land-cover, *Met*. Meteorological

Through the ensemble RF-ZIP model, the significant variables (p-value < 0.05) were identified for districts reporting API ≥ 0. The RRs estimated for these significant variables are provided in Fig. 5. The ensemble model indicates that the scheduled castes and Scheduled Tribes are among the most vulnerable sections as their percentage total are significantly associated [RR: 1.1 (95% CI 1.0, 1.13)] with the malaria transmission observed across the districts. Further, malaria transmission in the districts is also weakly associated with the percentage of people using unprotected water for household purposes [RR: 1.03 (95% CI 1.02, 1.04)]. In Ethiopia, unprotected source of drinking water was associated with increased malaria risk [32]. The fraction of the population travelling to collect water for household consumption is relatively strongly associated with increased transmission [RR: 1.2 (95% CI 1.17, 1.23)]. Travelling was identified as a significant risk factor for malaria transmission in Africa [33, 34].

In addition, the estimated associations based on the district-level malaria transmission for percentage of people not owning a mobile, signifying low socio-economic status and percentage of those who drink alcohol are 1.32 (95% CI 1.28, 1.37) and 1.07 (95% CI 1.04, 1.1), respectively. Alcohol consumption was previously associated as a risk factor in multiple studies [33, 34]. India, among the fastest growing economies in the world [35], has been witnessing a digital revolution but this is mostly restricted to urban and semi-urban areas, as reportedly only 29.3% of the population in rural India had broadband connections [36]. The government should focus on upgrading the mobile infrastructure in the highly endemic districts and use it for mass campaigns focused on malaria interventions and healthy lifestyle choices. Recently, the efficiency of mobile-based mass campaigns in reducing COVID-19 burden has been well documented [37–39]. The Malaria Elimination Demonstration Project in the Mandla district (Madhya Pradesh, India) relied on mobiles for surveillance-related activities and was successful in reducing the indigenous cases by about 91% [40].

Houses with muddy walls are among the significant determinants [RR: 1.06 (95% CI 1.02, 1.1] for increased malaria transmission in the endemic districts, suggesting a higher likelihood of malaria among the poorer sections of society. Regionally known as kutcha houses, they were associated with an increased likelihood of malaria cases in Mandla (India) [11], Baringo County, Kenya [41] and in Uganda [42]. In muddy houses, cracks may occur post-rain despite re-layering by inhabitants, which can become the breeding site for mosquitoes. Further, these houses commonly have thatched paddy as roof material, known for its water retention capacity, thus providing an active site for breeding. Substituting these with metal or cement could decrease the risk by 32% (95% CI 25, 39), which was observed on including the variable in the model. In the Gambia, a sub-Saharan African country experiencing a gradual decline in malaria in the past few decades, similarly to India, houses with metal roofs were avoided by *Anopheles* mosquitoes due to high temperatures observed inside the houses during the day, while their survival period was also reduced before they could become infectious [43].

It was observed that districts reporting high malaria cases are positively associated with maximum specific humidity and flooded vegetation, as the estimated RR was 1.07 (95% CI 1.03, 1.11) and 1.02 (95% CI 1.02, 1.03), respectively. Humidity is higher in forested areas or areas with significant green cover due to the greater ability of the soil to retain moisture because of the abundance of shade provided by trees [44]. Some 90% of the districts with high transmission in 2018 had very high forest cover and grasslands with considerable humidity. The ecosystems in forests significantly contribute to the global malaria burden [45], with control a major challenge [46, 47]. Humidity was also established as a critical parameter for mosquito control and transmission in The Gambia [48], India [46, 47] and Africa [46, 47]. Similarly, flooded vegetation provides sufficient breeding sites for transmission, as reported in Ghana [49]. Increased risk of malaria was associated with an increase in agricultural cover in the Democratic Republic of the Congo [50] and Belize [51].

The percentage of mothers with higher education levels was negatively associated (RR: 0.66 (95% CI 0.59, 0.74) with malaria transmission in the districts, suggesting the significance of maternal education, which is shown to be associated with decreased malaria risk in Africa [52]. In the high transmission districts (API > 5), a significant proportion (range: 10.4–64.9%) of mothers had no education. Supporting formal and informal education, especially in high-endemic districts, can help reduce the burden through increased awareness and informed choices on malaria prevention and control. There was a negative association between built-up areas and malaria risk in the districts with RR of 0.74 (95% CI 0.67, 0.8), suggesting that malaria transmission is higher in districts having fewer built-up areas, as also reported in Burkina Faso [53].

These findings are consistent with those observed for districts reporting zero API from the zero-inflated (logistic regression) component of the ZIP. For instance, the transmission is more likely to occur in the districts if the people are from scheduled castes and Scheduled Tribes (OR: 0.26, 95% CI 0.16, 0.41). An increase in flooded vegetation areas was associated with a decreased likelihood of zero transmission (OR: 0.04, 95% CI 0.005, 0.25). Alcohol consumption decreases the likelihood of zero transmission (OR: 0.83, 95% CI 0.66, 1.05). Both muddy walls (OR: 0.09, 95% CI 0.04, 0.2) and people travelling for collecting water (OR: 0.59, 95% CI 0.37, 0.92) increase the chances of transmission.

It was also observed that the percentage of mothers in higher education (OR: 0.19, 95% CI 0.1, 0.33) is associated with a decreased likelihood of zero transmission in the districts, suggesting that testing for malaria may increase with pre-existing knowledge and access to information when symptoms are observed. The categorical variable transmission was not significant for these districts due to a very low % *P. falciparum* present, but it was significant for all the districts with an estimated RR of 16.3 (95% CI 14.7, 18.0), indicating the role of *P. falciparum* in driving the transmission in the hotspots (Fig. 3).

**Fig. 6:**
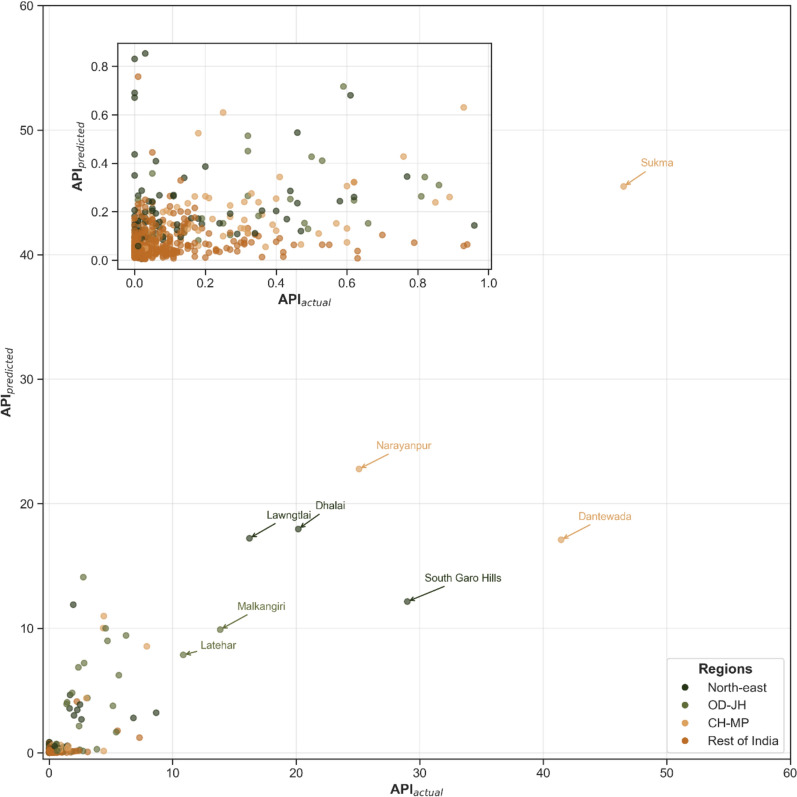
Comparison of actual vs predicted API across the districts

The model reported no heteroscedasticity, with a low RMSE (1.58) reported in post-regression diagnostic tests. Figure 6, representing the comparison of the actual and predicted API for the districts, indicates that the model could identify significant covariates influencing transmission across the districts while predicting well within acceptable ranges. However, the model is overestimating a few of the low-transmission districts. It is also underestimated in states such as Meghalaya, South Garo Hills. Upon its removal, the RMSE was reduced by 13.3%, but there was no significant change in the estimated associations. This may be attributed to the different sources of data as well as collection methods, while the northeastern states have vast geographical and cultural differences, which make them unique while also sharing international borders.

## Conclusions

The current study seeks to explain why some districts in a few states report very high transmission. Significant clusters of overall malaria transmission as well as *P. falciparum*-driven transmission were identified. Important potential drivers of the transmission were examined, indicating that people belonging to socio-economically marginalized communities continue to be the most vulnerable. People travelling to collect water, especially in forested areas, increase their risk of contracting malaria. Improving water accessibility should be a focus, along with education in high-risk areas and technological development to enhance awareness, thereby aiding the reduction of malaria cases and supporting central government’s goal of eliminating malaria by 2030. The identified clusters may be used as a perimeter for implementing targeted interventions specific to the entire cluster instead of a particular district, thus avoiding potential malaria spillover to neighbouring non-endemic districts. In contrast to recent legislation, i.e., the National Framework for Malaria Elimination, that uses districts as the focal point for interventions, these clusters are recommended as strategic points. Socio-economic development will further help reduce and eliminate the burden of malaria. The Government of India’s flagship Pradhan Mantri Gramin Awas Yojana, which is aimed at converting kutcha houses (e.g., muddy-walled houses) into pucca houses (e.g., bricked houses) in rural areas, is a positive approach towards the collective effort of improving socio-economic status. Inundated agricultural lands should be monitored for potential breeding sites, while localized outreach campaigns through multilateral communication models should be periodically organized to educate and inspire people to adopt self-protective measures against malaria and volunteer in community-based intervention methods.

The present study is limited by annual data which prevents from assessing the true impact of seasonality. Future work must focus on identifying the malaria risk by developing regional models over the years and predictive models at smaller spatiotemporal scales for the prevention of outbreaks. The present work uses an ecological study design, which is useful in understanding the drivers of the population-based patterns, especially in a diverse and heterogeneous country such as India. Further, the determinants identified here are recommended to be included in future longitudinal and cross-sectional studies at smaller spatial settings to assess the true causality. This can aid in minimizing the burden, and collectively working towards malaria elimination.

## Supplementary Information

Supplementary Material 1.

## Data Availability

The study utilised publicly accessible disease data from the National Center for Vector Borne Disease Control Programme. However, the curated data that support the findings of this study are available from the corresponding author, H.C.P., upon request.
