# Supplementary material for: Determinants of malaria transmission in Indian districts in 2018: insights from ensemble models
Source: Malar J. 2025 Oct 14;24:336. doi: 10.1186/s12936-025-05546-9 (PMC12522772; doi:10.1186/s12936-025-05546-9)
Supplement: Supplementary file 1 — Supplementary Material 1. [file 12936_2025_5546_MOESM1_ESM.docx]

*Supplementary Material on*

**Determinants of Malaria Transmission in Indian Districts in 2018: Insights from Ensemble Models**

**Avik Kumar Sam**

Environmental Science and Engineering Department, Indian Institute of Technology Bombay, Mumbai, India

**Alisha Khatoon**

Environmental Science and Engineering Department, Indian Institute of Technology Bombay, Mumbai, India

**Harish C. Phuleria**

Environmental Science and Engineering Department, Indian Institute of Technology Bombay, Mumbai, India

Center for Climate Studies, Indian Institute of Technology Bombay, Mumbai, India

National Disease Modelling Consortium, Indian Institute of Technology Bombay, Mumbai, India

Corresponding author: Harish C. Phuleria, [phuleria@iitb.ac.in](mailto:phuleria@iitb.ac.in)

**S1: Sensitivity Analysis**

The ensemble-ZIP model was also used using data from districts reporting API < 10. We observed that the overall RMSE reduced by 34.2%, and the model performed reasonably well in Chhattisgarh, Odisha, Madhya Pradesh, Mizoram and Telangana. However, for the Tripura, Manipur and Meghalaya, the model is generally overestimating the API. Districts having higher communities from scheduled castes and scheduled tribes, people living in muddy walled houses or those not owning a mobile are among the significant covariates. People who travel to get water were also positively associated with increased malaria transmission. In contrast to the overall model, people using an unprotected water source were negatively associated, indicating its importance in high-endemic areas. Covariates such as built areas, mothers having higher education and a tobacco-free lifestyle were negatively associated, similar to that observed for the overall model. The remaining covariates, such as alcohol consumption, flooded vegetation area, and maximum specific humidity, were not significant.

**Supplementary Figures & Tables:**


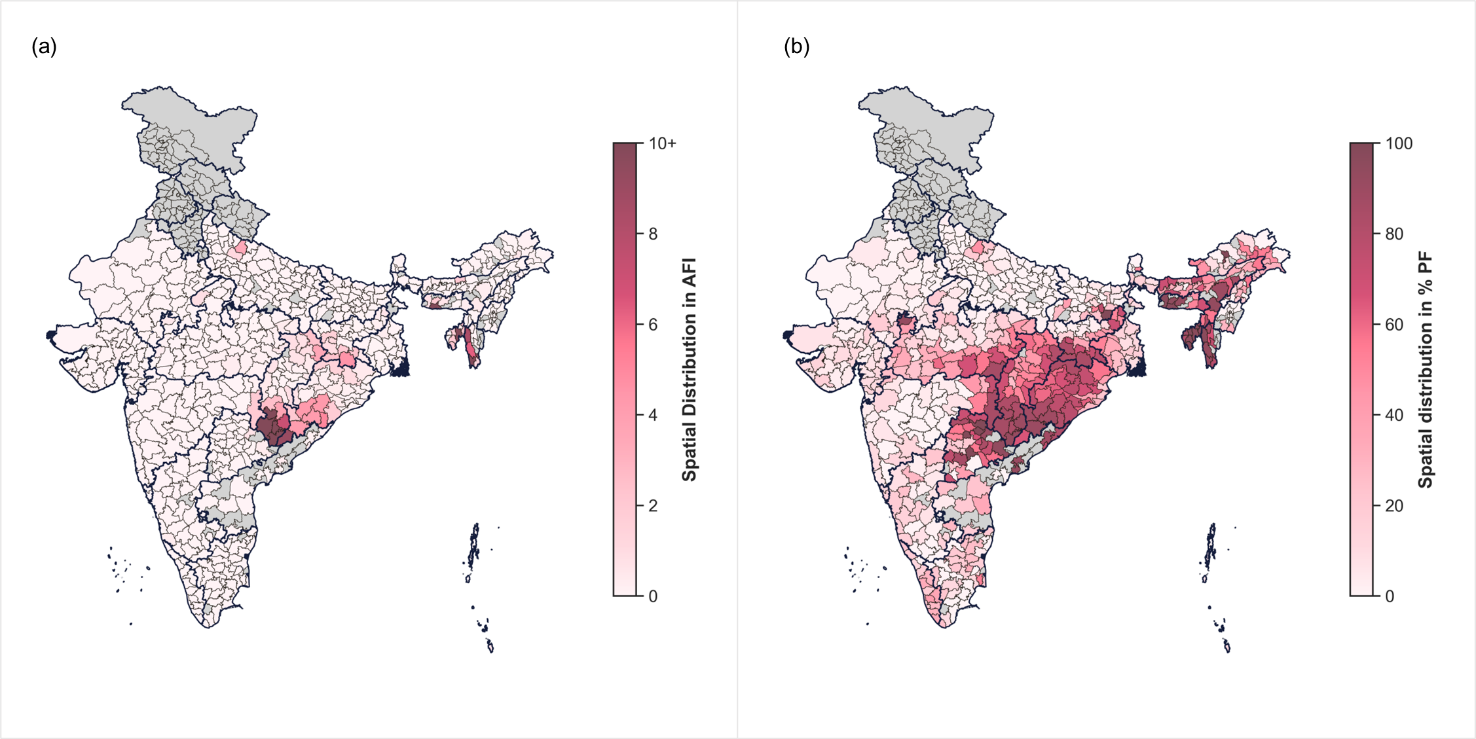


Figure S1 Spatial distribution of (a) AFI and (b) % PF across the districts.


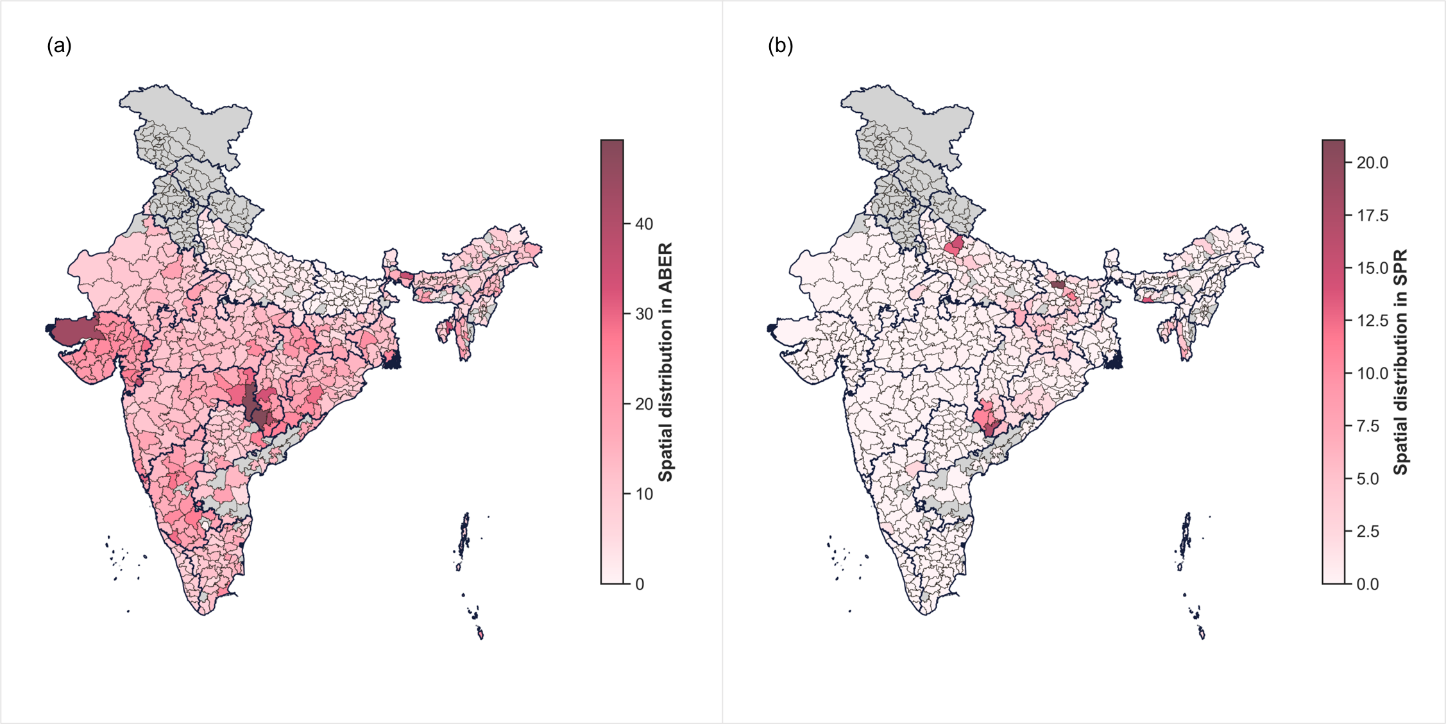


Figure S2 Spatial distribution of (a) ABER and (b) SPR across the districts.


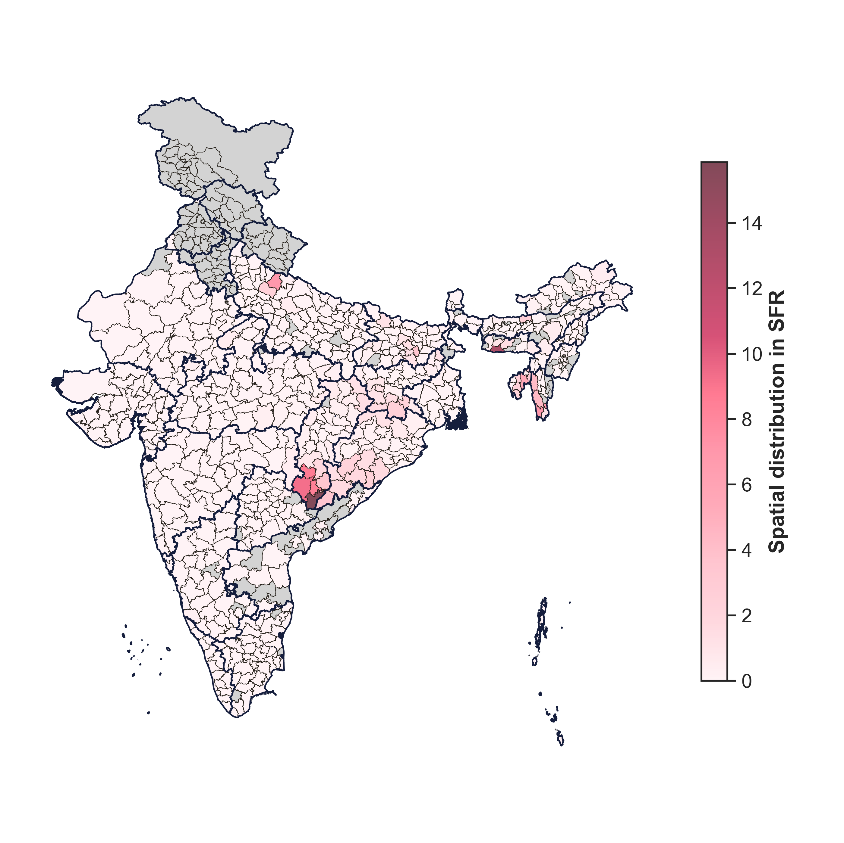


Figure S3 Spatial distribution of SFR across the districts.


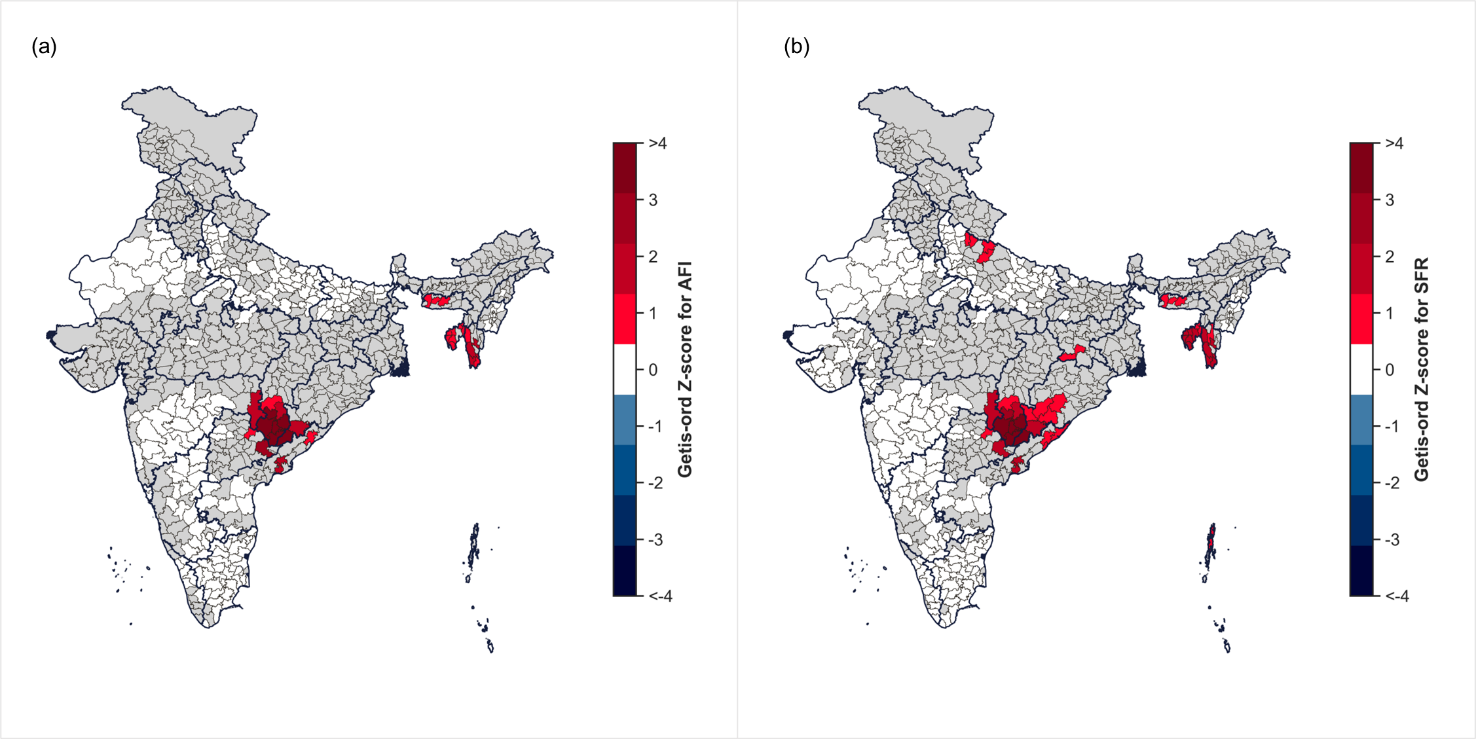


Figure S4 Spatial distribution in hotspots for (a) AFI and (b) SFR.


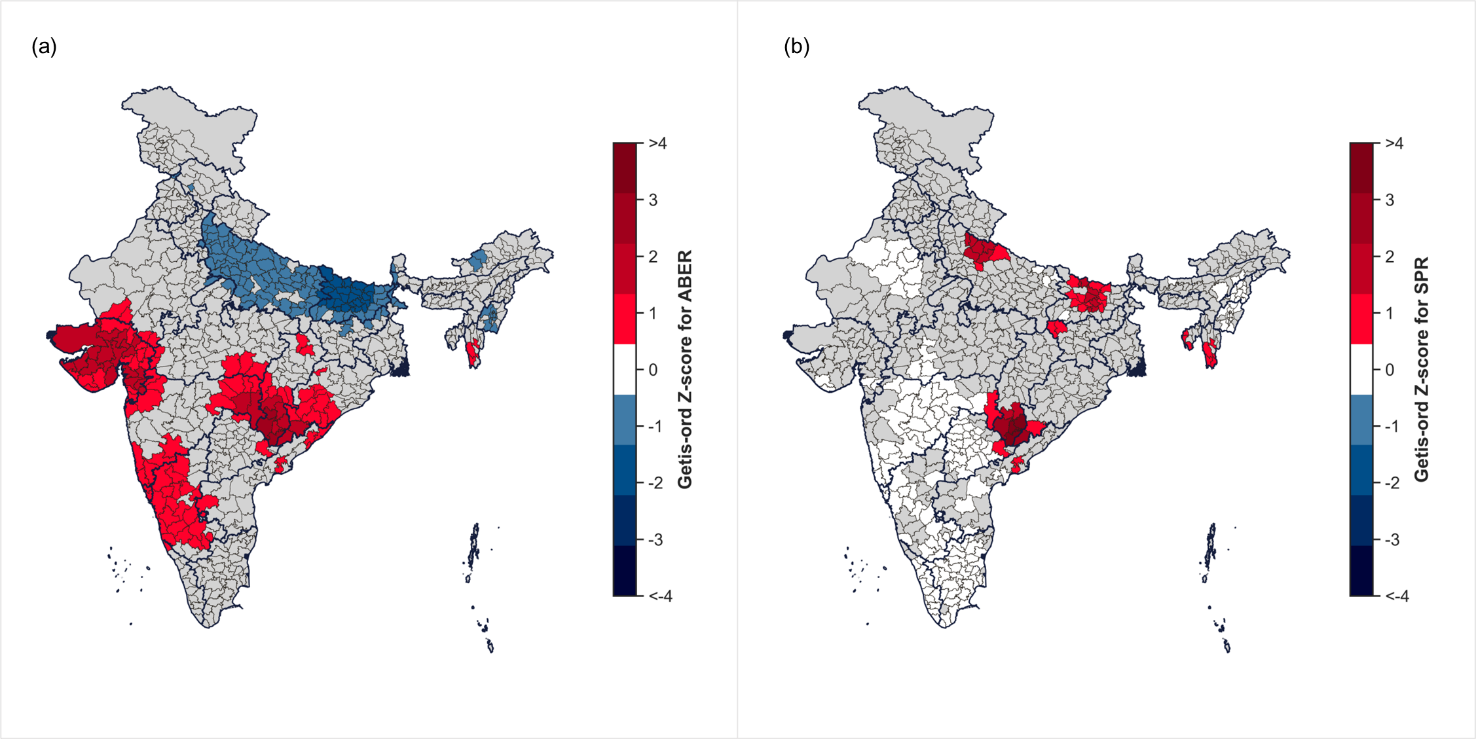


Figure S5 Spatial distribution in hotspots for (a) ABER and (b) SPR.


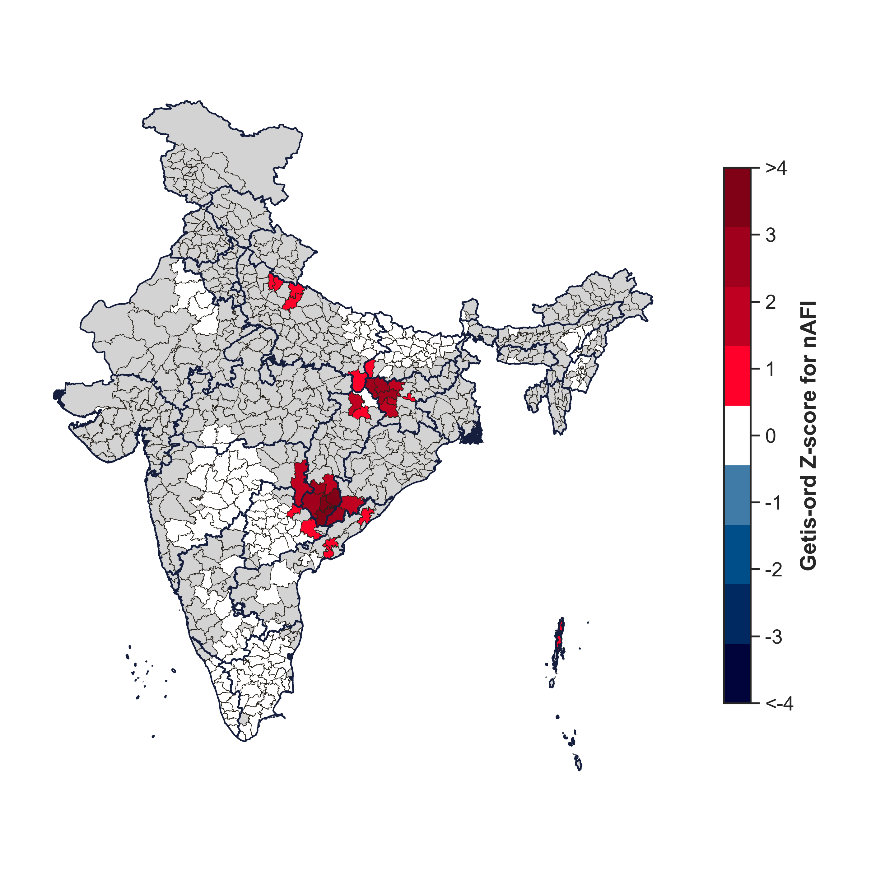


Figure S6 Spatial distribution in hotspots for non-AFI transmission.

Table S1 Distribution of the few variables considered for the ensemble modelling

| **Covariates** | **Min** | **Max** |
| --- | --- | --- |
| Did not sleep under a mosquito net | 3.81 | 100 |
| Doesn’t own a net | 0.45 | 99.78 |
| Hindu religion | 0 | 99.9 |
| Have disability | 0.55 | 9.29 |
| Backward Communities | 1.08 | 100 |
| Have Below Poverty Line Card | 5.78 | 96.85 |
| Average Age of Household Members | 20.95 | 45.8 |
| Electricity Access | 65.62 | 100 |
| Have Radio | 0.1 | 59.71 |
| Have television | 2.42 | 83.67 |
| Have refrigerator | 9.46 | 98.27 |
| Walls of Cement & concrete | 3.29 | 96.06 |
| Walls of Mud | 0 | 80.29 |
| Has mobile | 0.78 | 33.62 |
| Has computer | 56.85 | 99.24 |
| Owns livestock | 0.68 | 94.81 |
| Has internet access | 8.02 | 89.8 |
| DW piped | 0.63 | 95.46 |
| DW unprotected | 0 | 34.69 |
| Water source location elsewhere | 0 | 93.16 |
| No education in mothers | 0 | 67.34 |
| Higher education in mothers | 1.12 | 68.9 |
| Smoking | 34.9 | 98.8 |
| Alcohol consumption | 0.33 | 40.69 |
| Previous Blood Pressure Checked | 9.65 | 80.09 |
| Previous Blood Glucose Checked | 1.64 | 79.75 |
| Mean Specific Humidity (SH) | 0 | 0.03 |
| Max SH | 0.01 | 0.05 |
| Min SH | 0 | 0.02 |
| Bare ground | 0 | 10934 |
| Built Area | 0 | 1280.54 |
| Crops | 0 | 11879.5 |
| Flooded vegetation | 0 | 139.08 |
| Rangeland | 0 | 22981.5 |
| Trees | 0 | 7419.46 |
| Water | 0 | 2232.03 |
| Clouds | 0 | 71.47 |
| Snow/Ice | 0 | 2572.51 |
